# Drug-Coated Balloon Percutaneous Coronary Intervention in Diabetic and Non-Diabetic Patients: A Large All-Comers Cohort of Mid-Term Outcomes and Predictors of Adverse Events

**DOI:** 10.3390/jcm15145646

**Published:** 2026-07-18

**Authors:** Alessandro Sticchi, Alberto Cereda, Matteo Rocchetti, Mauro Gitto, Pier Pasquale Leone, Francesco Gioia, Alessia Latini, Francesco Tartaglia, Mauro Chiarito, Marco Luciano Rossi, Ottavia Cozzi, Gabriele Gasparini, Gianluigi Condorelli, Bernhard Reimers, Damiano Regazzoli, Giulio Giuseppe Stefanini, Antonio Mangieri, Antonio Colombo

**Affiliations:** 1 University of Pisa, 56126 Pisa, Italy; 2 IRCCS Humanitas Research Hospital, 20089 Rozzano, Italy; 3 EMO-GVM Centro Cuore Columbus, 20145 Milan, Italy; 4 Cardiovascular Department, ASST Santi Paolo e Carlo, 20122 Milan, Italy; 5 Università degli Studi di Milano, 20122 Milan, Italy; 6 The Zena and Michael A. Wiener Cardiovascular Institute, Icahn School of Medicine at Mount Sinai, New York, NY 10029, USA

**Keywords:** drug-coated balloon, diabetes mellitus, percutaneous coronary intervention, target lesion revascularisation, major adverse cardiovascular events

## Abstract

**Background:** Diabetes accelerates atherosclerosis and restenosis and impairs vascular healing, complicating percutaneous coronary intervention (PCI). Drug-coated balloons (DCBs) deliver antiproliferative therapy without a permanent scaffold, but their comparative mid-term performance in diabetic patients relative to non-diabetic patients is incompletely defined. **Methods:** We analysed a large all-comers cohort of consecutive patients who underwent a DCB-based PCI between 2018 and 2022, stratified by diabetes status. The primary endpoint was a composite of cardiac death, target vessel revascularisation (TVR) and target vessel myocardial infarction. Cumulative incidence was estimated by Kaplan–Meier analysis, and predictors were assessed by Cox regression. **Results:** Among 853 patients (304 diabetic, 549 non-diabetic), diabetic patients had more comorbidities and more frequent in-stent restenosis (52.3% vs. 41.8%). Over a median follow-up of 376 days, the two-year incidence of the composite endpoint was higher among diabetic patients (21.3% vs. 10.6%, *p* = 0.007), as was target lesion revascularisation (TLR; 13.6% vs. 5.5%, *p* = 0.004). Diabetes independently predicted the composite endpoint (adjusted hazard ratio: 1.87; 95% CI: 1.10–3.17) and TLR (adjusted HR: 2.33; 95% CI: 1.18–4.62), whereas DCB type and procedural variables did not. **Conclusions:** In an all-comers DCB-PCI population, diabetes was independently associated with higher rates of mid-term major adverse cardiovascular events and target lesion revascularisation, while device and procedural variables did not drive outcomes, underscoring the importance of systemic risk management in regard to diabetic patients.

## 1. Introduction

Diabetic patients have long been recognised as a complex group for percutaneous coronary revascularisation, often referred to as the “Achilles heel” of interventional cardiology. The pathophysiology of diabetes, characterised by accelerated atherosclerosis, chronic inflammation, endothelial dysfunction and delayed stent endothelialisation, complicates coronary interventions. Diabetic patients also exhibit an abnormal vascular response, with increased smooth muscle cell proliferation and a higher rate of restenosis [1].

Coronary disease in diabetic patients is frequently multivessel and complex. Surgical revascularisation, particularly coronary artery bypass grafting (CABG), has consistently demonstrated superior long-term outcomes compared with PCI, with a reduced risk of restenosis and more durable revascularisation [2]. The landmark FREEDOM trial showed that CABG significantly reduced major adverse cardiovascular events (MACEs), including cardiovascular mortality, myocardial infarction and the need for repeat revascularisation, although it carried a higher risk of stroke than PCI [3].

Since FREEDOM, PCI techniques have advanced considerably [4]. Attempts to surpass third-generation drug-eluting stents (DESs) with newer DES technology have not produced a major improvement [5], although progress in regard to antiplatelet, antithrombotic, and lipid-lowering therapies; novel antidiabetic agents with a proven cardiovascular benefit; and intracoronary imaging such as optical coherence tomography and intravascular ultrasound has further refined revascularisation strategies [6,7,8,9].

A promising approach in this context is the use of DCBs [10]. DCBs deliver antiproliferative drugs directly into the vessel wall without leaving a permanent metallic scaffold. While historically recommended for in-stent restenosis, their application in broader coronary settings is expanding [11]. Contemporary DCBs incorporating sirolimus and controlled drug-elution platforms are generally considered to offer improved safety and efficacy [12], and their versatility makes them well suited to the small-vessel disease and bifurcations frequently encountered in diabetic patients. Given the higher risk of restenosis and stent failure in diabetes, DCBs represent an attractive option for a more physiological, metal-free revascularisation [10].

DCBs are increasingly applied across heterogeneous lesion subsets, from their classic indication of in-stent restenosis to de novo disease and small vessels, and they are frequently combined with a stent in a hybrid strategy for long or complex lesions. How a DCB-based approach performs across this spectrum, along with which patient and lesion factors drive mid-term outcomes, remains incompletely characterised, especially in diabetic patients in whom insulin treatment often signals more advanced disease.

The aim of this study was to evaluate a DCB-based revascularisation strategy in diabetic and non-diabetic patients, in a large unselected all-comers cohort, in terms of survival, MACE, and their individual components over a mid-term follow-up, with the objective of clarifying the impact of diabetes on outcomes and identifying predictors of adverse events.

## 2. Materials and Methods

### 2.1. Study Design and Population

This was a retrospective analysis of consecutive patients undergoing DCB-based PCI between 2018 and 2022 at tertiary centres dedicated to the treatment of coronary artery disease (IRCCS Humanitas Research Hospital, Rozzano, and EMO-GVM Centro Cuore Columbus, Milan, Italy). All consecutive patients treated with at least one coronary DCB were eligible, without restriction by clinical presentation or lesion subset (an all-comers design). Patients were stratified by diabetes status into diabetic and non-diabetic groups according to the clinical diagnosis recorded in the institutional registry, as defined by a glycated haemoglobin level ≥ 6.5% and/or ongoing antidiabetic therapy. Diabetes subtype was not separately recorded; given the all-comers coronary population (mean age: approximately 71 years), the diabetic group was expected to comprise essentially exclusively type 2 diabetes patients.

### 2.2. Procedures

PCI was performed by experienced operators. DCB use followed the recommendations of the international DCB consensus [12]: adequate predilatation and lesion preparation were performed in all cases, and the DCBs were inflated for approximately 60 s. Both sirolimus- and paclitaxel-coated DCBs were used. A bailout stenting strategy was applied for residual stenosis greater than 30% or flow-limiting dissection, and a hybrid strategy combining a proximal DES with a distal DCB was adopted for long or complex lesions when required [11,13,14,15].

### 2.3. Endpoints and Follow-Up

The primary endpoint was a composite of cardiac death, TVR, and TVMI; TVR included TLR. Individual components and all-cause death were secondary endpoints. Clinical follow-up data were obtained, with event dates recorded; mortality data were derived from administrative databases when patients were not hospitalised. Time-to-event analyses included the 638 patients (74.8%) with an available follow-up time, and the median follow-up is reported. The unavailability of follow-up time in this study was predominantly an administrative and calendar-driven (registry records without a structured follow-up date and patients enrolled late in the study period) issue rather than true clinical loss to follow-up and did not differ by diabetes status (76.6% vs. 73.8%, *p* = 0.35).

### 2.4. Statistical Analysis

Continuous variables are presented as means ± standard deviations with the independent-samples *t*-test when approximately normally distributed or as medians [interquartile range] with the Mann–Whitney U test when skewed. Categorical variables are presented as n (%) and were compared using the chi-square test or Fisher’s exact test when expected counts were below five; percentages were computed using non-missing observations, and missing data were not imputed. Cumulative incidence was estimated using the Kaplan–Meier method and compared between groups using the log-rank test, with administrative censoring at 730 days for two-year estimates. Univariable and multivariable Cox proportional-hazards models were used to assess predictors of the composite endpoint, with results expressed as hazard ratios (HRs) and 95% confidence intervals (CIs); the multivariable model was adjusted for age, chronic kidney disease, in-stent restenosis and acute coronary syndrome at presentation. For target lesion revascularisation, given the limited number of events, a parsimonious multivariable Cox model adjusting for in-stent restenosis was fitted, with consistency checked in sensitivity analyses adjusting for chronic kidney disease or age. Missingness was examined in relation to diabetes status; it was comparable between groups for most variables and higher among non-diabetic patients for dyslipidaemia and selected procedural variables, whereas the covariates used in the multivariable models had minimal, non-differential missingness. Predefined subgroups defined by lesion type, balloon coating, hybrid strategy and insulin treatment were explored, and they were considered hypothesis-generating given the limited number of events. A two-sided *p* value below 0.05 was considered significant. Analyses were performed in Python 3 (scipy and lifelines).

## 3. Results

### 3.1. Study Population

From 863 registry records, 5 duplicate patient records and 5 patients with undetermined diabetes status were excluded, leaving a final analytic cohort of 853 patients: 304 diabetic (35.6%) and 549 non-diabetic (64.4%). Baseline clinical characteristics are summarised in Table 1. Diabetic patients were older (mean 71.7 vs. 69.2 years, *p* < 0.001) and had higher rates of hypertension (85.5% vs. 71.7%), dyslipidaemia (88.6% vs. 74.7%), chronic kidney disease (28.9% vs. 12.2%), previous myocardial infarction (61.8% vs. 44.2%), previous PCI (76.3% vs. 65.9%) and previous CABG (12.8% vs. 7.4%) (all *p* < 0.01). The proportion of women (about 16%) and the rate of acute coronary syndrome at presentation were similar between groups. Among diabetic patients, 23.4% were treated with insulin.

### 3.2. Procedural Characteristics

Procedural and angiographic characteristics are reported in Table 2. In-stent restenosis lesions were more frequent in diabetic patients (52.3% vs. 41.8%, *p* = 0.003), whereas a hybrid DCB-plus-DES strategy was used in a similar proportion in both groups (about 44%). Sirolimus-coated DCBs predominated and were used in approximately 79% of patients in both groups, and the quantities of DCBs per patient were comparable. Lesion preparation was performed in the large majority of cases, with high rates of predilatation and frequent use of non-compliant balloons; the DCB diameter was marginally larger in diabetic patients (median 3.0 vs. 2.8 mm, *p* = 0.018).

### 3.3. Clinical Outcomes

**Figure 1:**
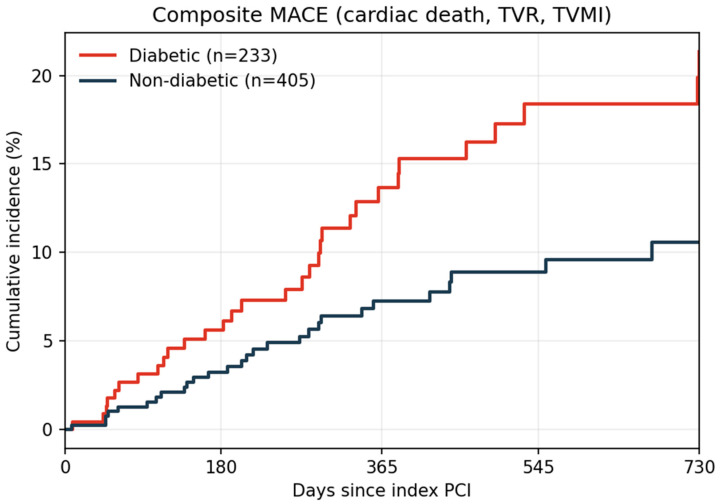
Kaplan–Meier cumulative incidence of the composite endpoint (cardiac death, target vessel revascularisation, or target vessel myocardial infarction) in diabetic versus non-diabetic patients. Survival cohort with available follow-up data (n = 638: 233 diabetic, 405 non-diabetic).

**Figure 2:**
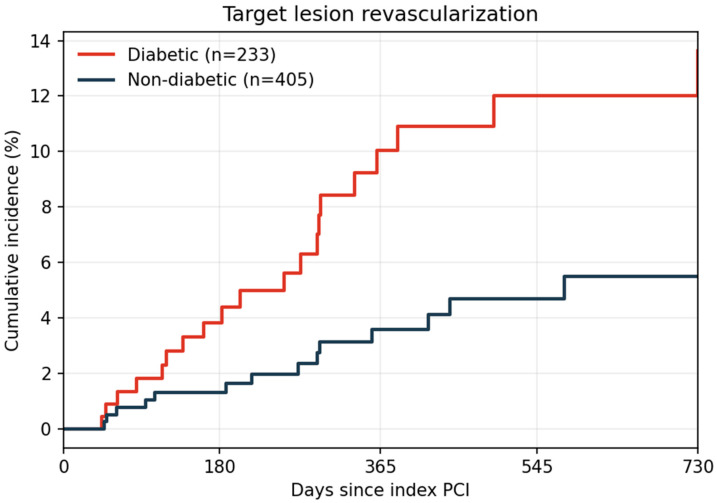
Kaplan–Meier cumulative incidence of target lesion revascularisation in diabetic versus non-diabetic patients. Survival cohort with available follow-up data (n = 638: 233 diabetic, 405 non-diabetic).

Follow-up time was available for 638 patients, with a median follow-up of 376 days. Cumulative incidence estimates at one and two years are reported in Table 3, and Kaplan–Meier curves for the composite endpoint and for TLR are shown in Figure 1 and Figure 2. At two years, diabetic patients had a significantly higher cumulative incidence of the composite endpoint (21.3% vs. 10.6%, *p* = 0.007), TVR including TLR (19.9% vs. 10.5%, *p* = 0.012), and TLR (13.6% vs. 5.5%, *p* = 0.004). Target-vessel-only revascularisation, TVMI, cardiac death and all-cause death did not differ significantly between groups, although the event numbers for TVMI and cardiac death were small.

### 3.4. Predictors of the Composite Endpoint

**Figure 3:**
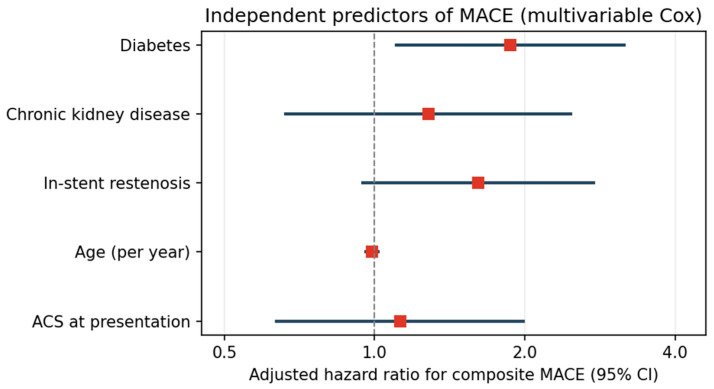
Independent predictors of the composite endpoint (multivariable Cox model). Squares denote adjusted hazard ratios, and horizontal lines indicate the 95% confidence intervals.

Univariable and multivariable predictors of the composite endpoint are reported in Table 4, and the independent predictors are displayed in Figure 3. According to univariable analysis, diabetes (HR 2.00, 95% CI 1.20–3.34, *p* = 0.008), serum creatinine (HR 1.28 per mg/dL, 95% CI 1.08–1.51, *p* = 0.005) and in-stent restenosis (HR 1.73, 95% CI 1.02–2.93, *p* = 0.043) were associated with a higher risk, whereas a hybrid strategy was associated with a lower risk (HR 0.54, 95% CI 0.31–0.95, *p* = 0.032); balloon coating, vessel size and the remaining variables were not significant. After multivariable adjustment, diabetes remained an independent predictor of the composite endpoint (HR 1.87, 95% CI 1.10–3.17, *p* = 0.020), with in-stent restenosis showing a borderline association (HR 1.61, 95% CI 0.95–2.74, *p* = 0.078). In an unadjusted model, diabetes was associated with TLR (HR 2.60, 95% CI 1.32–5.16, *p* = 0.006); after adjustment for in-stent restenosis, it remained an independent predictor of TLR (adjusted HR 2.33, 95% CI 1.18–4.62, *p* = 0.015), with consistent estimates when adjusting for chronic kidney disease (HR 2.53, 95% CI 1.26–5.08) or age (HR 2.63, 95% CI 1.32–5.22) instead.

### 3.5. Lesion Type, Balloon Coating and Hybrid Strategy

**Figure 4:**
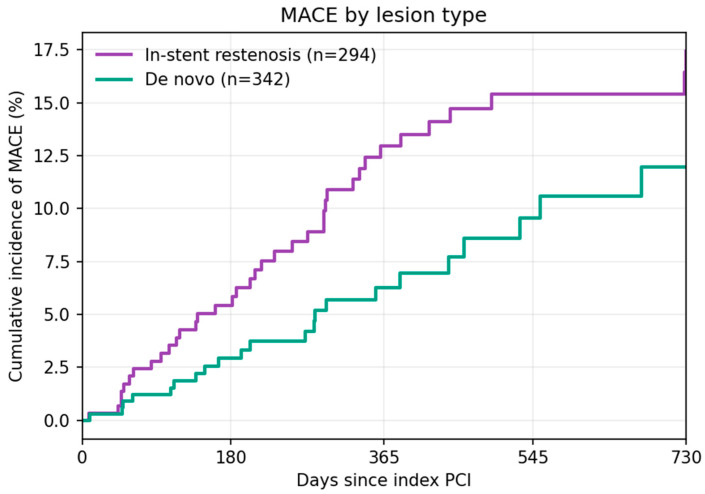
Kaplan–Meier cumulative incidence of the composite endpoint in in-stent restenosis versus de novo lesions. Survival cohort with documented lesion type (n = 636: 294 in-stent restenosis, 342 de novo; lesion type missing for 2 patients).

**Figure 5:**
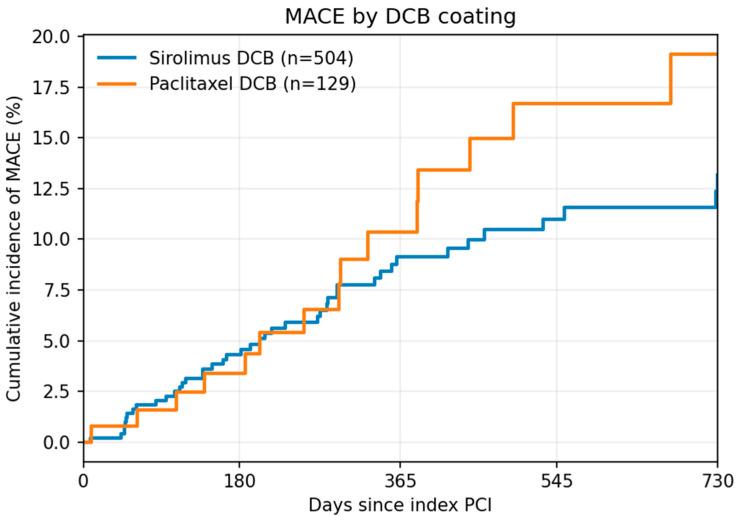
Kaplan–Meier cumulative incidence of the composite endpoint with sirolimus- versus paclitaxel-coated drug-coated balloons.

The results of exploratory subgroup analyses of the composite endpoint are shown in Figure 4 and Figure 5. Outcomes did not differ significantly between sirolimus- and paclitaxel-coated DCBs (two-year MACE: 13.2% vs. 19.1%, *p* = 0.314; TLR: 7.0% vs. 12.6%, *p* = 0.348), numerically favouring sirolimus. In-stent restenosis lesions carried a higher event burden than de novo lesions (two-year MACE: 17.5% vs. 12.0%, *p* = 0.040; TLR: 13.8% vs. 2.6%, *p* < 0.001). A hybrid DCB-plus-DES strategy was associated with fewer events than a DCB-only strategy (two-year MACE: 8.4% vs. 18.5%, *p* = 0.029; TLR: 2.6% vs. 12.3%, *p* < 0.001); however, this difference largely reflects confounding by indication, because DCB-only treatment was preferentially used for in-stent restenosis, which carries a higher recurrence risk. Given the small number of events, these subgroup analyses are hypothesis-generating.

### 3.6. Insulin-Treated Diabetic Patients

Among diabetic patients, 71 (23.4%) were treated with insulin. As shown in Table 5, insulin-treated diabetic patients had higher serum creatinine (median 1.1 vs. 0.9 mg/dL, *p* = 0.012), lower eGFR (65.2 vs. 75.4 mL/min/1.73 m2, *p* = 0.012), more chronic kidney disease (38.6% vs. 26.0%, *p* = 0.042), and more previous CABG (19.7% vs. 10.7%, *p* = 0.047) than non-insulin-treated diabetic patients, a finding consistent with a more advanced disease burden. Mid-term outcomes did not differ significantly between the two subgroups (two-year MACE: 18.4% vs. 21.9%, *p* = 0.594; TLR: 7.3% vs. 15.6%, *p* = 0.365), although event numbers were small.

## 4. Discussion

In this large all-comers cohort, a DCB-based PCI strategy was applied across the full spectrum of patients and lesions. The principal findings can be summarised as follows. First, diabetic patients experienced higher rates of the composite endpoint and of TLR than non-diabetic patients. Second, diabetes was an independent predictor of both the composite endpoint and TLR after multivariable adjustment, whereas DCB type and procedural variables were not. Third, the separation of the Kaplan–Meier curves emerged progressively over the follow-up, a finding consistent with a growing influence of systemic disease over time.

Beyond their established role in in-stent restenosis, DCBs have increasingly emerged as a viable option for treating de novo coronary lesions. By delivering antiproliferative therapy without a permanent scaffold, DCBs reduce long-term stent-related complications, an outcome consistent with the ‘leave-nothing-behind’ strategy. Evidence from our group and from recent randomised data has demonstrated outcomes comparable with DES while substantially reducing stent burden, supporting DCB use in selected de novo cases [11,13]. In the present cohort, nearly half of the procedures used a hybrid strategy, reflecting a deliberate, lesion-tailored approach in which a proximal DES and a distal DCB were combined in long or complex lesions to ensure complete revascularisation while limiting total stent burden, with similar outcomes between groups.

Taken together, these findings suggest that lesion-level success with DCB may be progressively offset by systemic drivers in diabetes, including endothelial dysfunction, chronic inflammation, microvascular disease and impaired healing. This pathophysiological substrate plausibly explains why patient-level factors dominate mid-term outcomes over device-related variables, in line with prior evidence on DCB use and diabetes risk profiles [16,17,18,19]. The complexity of managing coronary artery disease in diabetic patients, characterised by accelerated atherosclerosis and heightened inflammatory responses, is well documented [18]. The recent Academic Research Consortium position statement supports the progressive extension of DCB use beyond in-stent restenosis, particularly in small-vessel disease, diffuse lesions, and complex clinical scenarios such as diabetes or high bleeding risk, emphasising that the long-term benefit depends primarily on optimal lesion preparation, accurate vessel sizing and the absence of flow-limiting dissections rather than on device selection alone [12].

### 4.1. Comparison with Previous Evidence

Our findings are consistent with the BASKET-SMALL 2 trial and the PICCOLETO-II trial, which reported durable outcomes of DCB angioplasty in small vessels and indicated that long-term results are mainly driven by patient comorbidity rather than device technology [16,17]. They also align with observational data showing that diabetes and renal dysfunction remain independent predictors of adverse events after DCB angioplasty [19]. The comparable outcomes observed between sirolimus- and paclitaxel-coated DCBs in routine use are in keeping with the contemporary consensus, which attributes residual risk to vascular healing and patient biology rather than to a class-specific drug effect [12]. Insulin-treated diabetic patients, who tend to have more advanced disease and renal impairment, have previously been shown to experience worse outcomes after PCI, reinforcing the importance of comprehensive management for this subgroup [20].

Our subgroup findings refine this picture. The higher event rate of in-stent restenosis compared with de novo lesions, driven mainly by target lesion revascularisation, is consistent with the more recurrent biology of restenotic tissue and supports the expanding use of DCBs in de novo disease reported by our group and others [11,13]. The apparently lower event rate with a hybrid strategy should be interpreted cautiously because a DCB-only approach was preferentially chosen for in-stent restenosis, so this association largely reflects confounding by indication rather than genuine superiority of hybrid PCI. Insulin-treated diabetic patients showed the expected enrichment in renal impairment and prior surgical revascularisation, yet their mid-term event rates were not significantly different from those of non-insulin-treated diabetic patients in this underpowered comparison [20].

### 4.2. Clinical Implications

These results indicate that, in metabolically compromised patients, lesion-level optimisation alone cannot fully overcome systemic disease. A successful DCB-based PCI therefore relies on meticulous lesion preparation and integrated risk control rather than on balloon selection alone. Clinically, diabetic patients may benefit from a multidisciplinary approach combining strict metabolic and renal management, individualised antithrombotic therapy, and, when indicated, hybrid strategies that balance complete revascularisation with reduced stent burden and preserved vessel physiology.

### 4.3. Limitations

This study has several limitations. Its observational, retrospective design precludes causal inference and is subject to residual confounding despite multivariable adjustment. Follow-up times were available for 638 of the 853 patients, and time-to-event analyses were restricted to these patients, which may have introduced attrition bias. Availability of follow-up did not differ by diabetes status (76.6% vs. 73.8%, *p* = 0.35), forming the basis of an argument against differential attrition. The event numbers for some endpoints, particularly target vessel myocardial infarction and cardiac death, were small, so the corresponding comparisons are underpowered and their confidence intervals wide, and they should be interpreted with caution. Some procedural variables were incompletely recorded in the registry, and percentages were computed on available data without imputation. Missingness was largely comparable between diabetic and non-diabetic patients but higher among non-diabetic patients for dyslipidaemia and selected procedural variables, which may have affected the selected descriptive baseline estimates; the covariates used in the adjusted models had minimal, non-differential missingness. Diabetes was classified according to the status recorded in the registry rather than by centrally adjudicated glycated haemoglobin, and angiographic endpoints were site-reported without core-laboratory adjudication. Finally, the cohort reflects the practices of experienced operators at two dedicated DCB programmes, which may limit generalisability to lower-volume settings. Larger, prospective and adjudicated multicentre studies are required to confirm these findings.

## 5. Conclusions

In a large all-comers population undergoing DCB-based PCI, diabetes was independently associated with a higher mid-term risk of major adverse cardiovascular events and target lesion revascularisation, whereas DCB type and procedural variables did not drive outcomes. DCB-based PCI is feasible across the spectrum of patients, but mid-term prognosis in diabetic patients is governed by systemic disease. These data reinforce the importance of individualised, comprehensive risk management, with particular attention to renal function and glycaemic burden, in diabetic patients undergoing DCB-based revascularisation.

## Figures and Tables

**Table 1:** Baseline clinical characteristics.

Variable	Diabetic (n = 304)	Non-Diabetic (n = 549)	*p*	Missing
**Age, years**	71.7 ± 9.5	69.2 ± 10.5	<0.001	0
**Female sex**	50 (16.5)	84 (15.4)	0.685	6
**Body mass index, kg/m^2^ ^†^**	27.2 [24.8–30.4]	26.3 [24.2–28.7]	0.006	188
**Hypertension**	260 (85.5)	393 (71.7)	<0.001	1
**Dyslipidaemia**	248 (88.6)	349 (74.7)	<0.001	106
**Smoking (current or former)**	180 (59.2)	295 (53.7)	0.123	0
**Current smoking**	60 (19.7)	87 (15.8)	0.150	0
**Serum creatinine, mg/dL ^†^**	1.0 [0.8–1.3]	0.9 [0.8–1.0]	<0.001	5
**eGFR, mL/min/1.73 m^2^ ^†^**	79.6 [57.3–93.4]	88.8 [73.9–97.2]	<0.001	10
**Chronic kidney disease**	87 (28.9)	66 (12.2)	<0.001	10
**Dialysis**	8 (2.8)	4 (0.8)	0.036	64
**Atrial fibrillation**	33 (11.7)	48 (10.2)	0.519	103
**Oral anticoagulation**	58 (20.6)	82 (17.4)	0.277	102
**Previous myocardial infarction**	188 (61.8)	242 (44.2)	<0.001	1
**Previous PCI**	232 (76.3)	362 (65.9)	0.002	0
**Previous CABG**	39 (12.8)	40 (7.4)	0.009	5
**ACS at presentation**	73 (24.1)	113 (20.7)	0.246	3
**Insulin-treated (of diabetics)**	71 (23.4)	0 (0.0)	<0.001	0

Continuous variables marked ^†^ are non-normally distributed and reported as medians [IQR] with the Mann–Whitney U test; other continuous variables are reported as means ± SDs with the *t*-test. Categorical variables are reported as n (%). Percentages were computed using non-missing observations.

**Table 2:** Procedural and angiographic characteristics.

Variable	Diabetic (n = 304)	Non-Diabetic (n = 549)	*p*	Missing
**In-stent restenosis (vs. de novo)**	159 (52.3)	227 (41.8)	0.003	6
**Hybrid PCI (DCB + DES)**	133 (43.9)	248 (45.5)	0.651	5
**Number of DCB per patient ^†^**	1.0 [1–1]	1.0 [1–1]	0.956	7
**DCB diameter, mm ^†^**	3.0 [2–4]	2.8 [2–3]	0.018	11
**DCB length, mm ^†^**	30.0 [20–30]	20.0 [20–30]	0.195	12
**Sirolimus-coated DCB (vs. paclitaxel)**	239 (79.7)	428 (79.1)	0.849	12
**Predilatation**	284 (96.3)	476 (93.5)	0.098	49
**Non-compliant balloon**	194 (66.0)	315 (62.0)	0.26	51
**Cutting balloon**	22 (7.3)	61 (11.3)	0.063	9
**Scoring balloon**	7 (2.5)	1 (0.2)	0.006	107
**Rotational atherectomy**	4 (1.3)	22 (4.1)	0.028	9
**Intravascular lithotripsy**	7 (2.3)	22 (4.1)	0.183	9
**Intravascular imaging (IVUS/OCT)**	51 (17.3)	96 (18.1)	0.792	28

^†^ median [IQR], Mann–Whitney U test. IQR, interquartile range.

**Table 3:** Cumulative incidence of clinical events at one and two years (Kaplan–Meier estimates).

Endpoint	Diabetic 1-yr (%)	Diabetic 2-yr (%)	Non-Diabetic 1-yr (%)	Non-Diabetic 2-yr (%)	Log-Rank *p*
**Composite MACE**	13.6	21.3	7.2	10.6	0.007
**TVR (including TLR)**	12.9	19.9	6.4	10.5	0.012
**Target lesion revascularisation**	10.0	13.6	3.6	5.5	0.004
**Target-vessel revascularisation (vessel only)**	8.0	13.3	4.8	8.3	0.134
**Target vessel myocardial infarction**	0.5	0.5	1.5	2.1	0.225
**Cardiac death**	0.9	2.7	0.5	0.5	0.125
**All-cause death**	2.8	7.4	2.7	4.3	0.379

Composite MACE: cardiac death, target vessel revascularisation, or target vessel myocardial infarction. Estimates from the survival cohort with available follow-up data (n = 638: 233 diabetic, 405 non-diabetic; median follow-up: 376 days). MACE, major adverse cardiovascular event.

**Table 4:** Univariable and multivariable predictors of the composite endpoint.

Variable	Univariable HR (95% CI)	*p*	Multivariable HR (95% CI); *p*
**Diabetes**	2.00 (1.20–3.34)	0.008	1.87 (1.10–3.17); *p* = 0.020
**Age (per year)**	1.00 (0.97–1.02)	0.753	0.99 (0.96–1.01); *p* = 0.368
**Female sex**	1.34 (0.73–2.49)	0.348	
**Hypertension**	0.79 (0.44–1.40)	0.417	
**Dyslipidaemia**	1.34 (0.66–2.75)	0.421	
**Current smoking**	1.29 (0.71–2.36)	0.399	
**Chronic kidney disease**	1.42 (0.77–2.63)	0.263	1.28 (0.66–2.47); *p* = 0.459
**Serum creatinine (per mg/dL)**	1.28 (1.08–1.51)	0.005	
**Atrial fibrillation**	1.23 (0.56–2.73)	0.607	
**Previous myocardial infarction**	1.44 (0.84–2.45)	0.181	
**Previous PCI**	1.29 (0.71–2.35)	0.407	
**Previous CABG**	1.66 (0.79–3.50)	0.183	
**ACS at presentation**	1.19 (0.67–2.08)	0.555	1.12 (0.64–1.99); *p* = 0.686
**In-stent restenosis**	1.73 (1.02–2.93)	0.043	1.61 (0.95–2.74); *p* = 0.078
**Hybrid PCI**	0.54 (0.31–0.95)	0.032	
**Sirolimus DCB**	0.74 (0.41–1.33)	0.316	
**DCB diameter (per mm)**	1.05 (0.87–1.26)	0.614	
**Diabetes (TLR endpoint)**	2.60 (1.32–5.16)	0.006	2.33 (1.18–4.62); *p* = 0.015

Hazard ratios from Cox proportional-hazards models. The multivariable model included diabetes, age, chronic kidney disease, in-stent restenosis and acute coronary syndrome (60 events). The final row reports diabetes as a predictor of target lesion revascularisation (secondary endpoint); the multivariable TLR estimate is adjusted for in-stent restenosis (34 events). HR, hazard ratio; CI, confidence interval; DCB, drug-coated balloon; PCI, percutaneous coronary intervention; ACS, acute coronary syndrome.

**Table 5:** Insulin-treated versus non-insulin-treated diabetic patients.

Variable	Insulin-Treated (n = 71)	Non-Insulin (n = 233)	*p*
**Age, years**	71.1 ± 10.7	71.9 ± 9.1	0.607
**Female sex**	15 (21.4)	35 (15.0)	0.205
**Serum creatinine, mg/dL ^†^**	1.1 [0.9–1.6]	0.9 [0.8–1.2]	0.004
**eGFR, mL/min/1.73 m^2^**	65.2 ± 30.3	75.4 ± 25.1	0.012
**Chronic kidney disease**	27 (38.6)	60 (26.0)	0.042
**Previous CABG**	14 (19.7)	25 (10.7)	0.047
**Previous myocardial infarction**	48 (67.6)	140 (60.1)	0.253
**Hypertension**	59 (83.1)	201 (86.3)	0.507
**Dyslipidaemia**	58 (87.9)	190 (88.8)	0.84

^†^ median [IQR], Mann–Whitney U test; other continuous variables are presented as means ± SDs, *t*-test. Categorical variables are given as n (%). eGFR, estimated glomerular filtration rate; CABG, coronary artery bypass grafting.

## Data Availability

The data underlying this article are available from the corresponding author on reasonable request.
